# Ginkgolide B Inhibits JAM-A, Cx43, and VE-Cadherin Expression and Reduces Monocyte Transmigration in Oxidized LDL-Stimulated Human Umbilical Vein Endothelial Cells

**DOI:** 10.1155/2015/907926

**Published:** 2015-07-12

**Authors:** Xueqing Liu, Wenjia Sun, Yanyang Zhao, Beidong Chen, Wei Wu, Li Bao, Ruomei Qi

**Affiliations:** Beijing Institute of Geriatrics, Beijing Hospital and Key Laboratory of Geriatrics Ministry of Health, Beijing 100730, China

## Abstract

*Aim*. To investigate the effect of ginkgolide B on junction proteins and the reduction of monocyte migration in oxidized low-density lipoprotein- (ox-LDL-) treated endothelial cells. *Methods*. Human umbilical vein endothelial cells (HUVECs) were used in the present study. Immunofluorescence and Western blot were performed to determine the expression of junctional adhesion molecule-A (JAM-A), connexin 43 (Cx43), and vascular endothelial cadherin (VE-cadherin). Monocyte migration was detected by the Transwell assay. *Results*. ox-LDL stimulation increased JAM-A expression by 35%, Cx43 expression by 24%, and VE-cadherin expression by 37% in HUVECs. Ginkgolide B (0.2, 0.4, and 0.6 mg/mL) dose-dependently abolished the expression of these junction proteins. The monocyte transmigration experiments showed that the level of monocyte migration was sixfold higher in the ox-LDL-treated group than in the control group. Ginkgolide B (0.6 mg/mL) nearly completely abolished monocyte migration. Both ginkgolide B and LY294002 suppressed Akt phosphorylation and the expression of these junction proteins in ox-LDL-treated endothelial cells. These results suggest that the ginkgolide B-induced inhibition of junction protein expression is associated with blockade of the PI3K/Akt pathway. *Conclusion*. Ginkgolide B suppressed junction protein expression and reduced monocyte transmigration that was induced by ox-LDL. Ginkgolide B may improve vascular permeability in atherosclerosis.

## 1. Introduction

Atherosclerosis is a chronic inflammatory disease of the vessel wall that is related to endothelial cell injury, smooth muscle cell migration and proliferation, platelet activation, and leukocyte accumulation. Oxidized low-density lipoprotein (ox-LDL) is a product of oxidative stress under pathological conditions and a major risk factor in atherosclerosis. ox-LDL induces an inflammatory response in endothelial cells, promotes leukocyte transmigration into the subintimal space, and involves vascular inflammation [1, 2].

Endothelial cells are linked to each other by two types of junction proteins: adherens junctions (AJs) and tight junctions (TJs). Previous studies have reported that endothelial cells play a role in maintaining endothelial monolayer integrity. However, recent studies have indicated that endothelial cells play a role in regulating leukocyte transmigration under pathological conditions, such as atherosclerosis and oxidative stress, but the underlying mechanisms remain unclear.

Junctional adhesion molecule-A (JAM-A) is a transmembrane glycoprotein that belongs to the immunoglobulin superfamily and is selectively concentrated at the intercellular junction of endothelial and epithelial cells [3]. The JAM family is composed of three classic members: JAM-A, JAM-B, and JAM-C. Studies have indicated that JAM-A may play differential roles in cell-cell adhesion, leukocyte migration, and platelets activation [4, 5]. A recent study reported that ox-LDL stimulation induced JAM-A redistribution in endothelial cells and increased the transmigration of mononuclear cells [6].

Connexins (Cxs) are a large family of transmembrane proteins that form hemichannels, which in turn constitute the gap junction (GJ) channel. By serving as direct conduits, these channels play multiple roles in neighboring cell communication via the facilitation of signaling molecule diffusion. Four connexins are expressed in the vascular wall: Cx37, Cx40, Cx43, and Cx45. Connexins have several important biological functions, including regulation of vascular permeability in the development of atherosclerosis [7–10]. Growing evidence indicates that connexins are involved in inflammatory signaling, such as adhesion molecule expression and leukocyte transmigration [11, 12]. The gap junction protein Cx43 is enriched in endothelial cells where it may play various biological roles that are unrelated to classic intercellular communication [13, 14]. Isakson et al. recently reported that oxidized phospholipids increase Cx43 expression in endothelial cells, vascular smooth muscle cells, and atherosclerotic mice [15]. The downregulation of Cx43 expression can inhibit atherosclerotic lesion formation in LDL receptor-deficient mice [16].

Vascular endothelial cadherin (VE-cadherin) is specifically expressed in endothelial cells and localized to AJs. VE-cadherin is a calcium-dependent and homophilic cell-cell adhesion molecule. The surface expression of VE-cadherin is localized to cell-cell junctions and associates with *α*-catenin, *β*-catenin, plakoglobin (*γ*-catenin), and p120-catenin through its cytoplasmic tail and actin cytoskeleton [17, 18]. VE-cadherin has two tyrosine residues (Y658 and Y731) that can be phosphorylated though stimulation by vascular endothelial growth factor (VEGF), tumor necrosis factor-*α* (TNF-*α*), platelet-activating factor (PAF), and thrombin. Previous studies have indicated that the docking of leukocytes to endothelial cells can stimulate the tyrosine phosphorylation of VE-cadherin, resulting in destabilization of the AJ complex and an increase in monolayer permeability [19–21].

Ginkgolide B is an extract of the ginkgo leaf that antagonizes the PAF receptor by competitively binding to it. Ginkgolide B has recently been shown to have beneficial biological effects on the protection of endothelial cells [22–24]. We reported a decrease in the level of reactive oxygen species (ROS), nicotinamide adenine dinucleotide phosphate-oxidase NOX4, and intercellular adhesion molecule-1 (ICAM-1) in ox-LDL-stimulated HUVECs [25]. In the present study, we further investigated the effects of ginkgolide B on vascular permeability and junction protein expression in HUVECs.

## 2. Material and Methods

### 2.1. Ethics Statement

The present study was approved by the ethics committee of the Beijing Institute of Geriatrics (number 20100512). Informed consent was obtained according to the Declaration of Helsinki. Freshly isolated umbilical cords were obtained from healthy donors who provided written informed consent.

### 2.2. Materials

Ginkgolide B was purchased from Daguanyuan Company (Xuzhou, China) and had a purity of 95%. Anti-JAM-1 and anti-Cx43 antibodies were purchased from Santa Cruz Biotechnology (Santa Cruz, CA, USA). Anti-VE-cadherin antibody was purchased from Abcam (Boston, MA, USA). Dylight 488 affini Pure Coat-anti-rabbit antibodies were purchased from EarthOx (San Francisco, CA, USA). Anti-tyrosine phosphorylated antibody 4G10 was purchased from Millipore (Billerica, MA, USA). Anti-Akt and anti-phosphorylated Akt antibodies were purchased from Cell Signaling Technology (Danvers, MA, USA). LY294002 was purchased from Sigma-Aldrich (St. Louis, MO, USA). Transwell permeable supports were purchased from Corning (Lowell, MA, USA).

### 2.3. Preparation and Culture of HUVECs

Primary HUVECs were collected from the human umbilical vein via collagenase I digestion for 15 min. After washing in phosphate-buffered saline (PBS), the solution was collected and centrifuged at 1000 rotations per minute for 6 min. The cells were collected and cultured in M199 that contained 20% fetal bovine serum, 2 mM glutamine, and 1% penicillin/streptomycin in an incubator at 37°C with 5% CO_2_. HUVECs at passages 2–4 were used in the present study.

### 2.4. Cell and Western Blot Analysis

HUVECs were preincubated with various concentrations of ginkgolide B and LY294002 for 1 h. The cells were then exposed to ox-LDL for 6 h. Cell lysis was performed in lysis buffer (1% Triton X-100, 100 mM Tris/HCl [pH 7.2], 50 mM NaCl, 5 mM ethylenediaminetetraacetic acid, 5 mM ethylene glycol tetraacetic acid, 1 mM phenylmethylsulfonyl fluoride, and 100 *μ*g/mL leupeptin). Cellular protein concentrations were determined using the Coomassie-blue assay. Protein samples were boiled in sodium dodecyl sulfate (SDS) loading buffer, run on SDS-polyacrylamide gel, and transferred to a polyvinylidene difluoride membrane (Millipore). Primary antibody incubations were performed overnight at 4°C. Horseradish peroxidase-conjugated secondary antibodies were applied for 1 h at room temperature and developed using Super Signal developing reagent (Pierce, Thermo Scientific, Rockford, IL, USA). Blot densitometry was then performed, and the bands were analyzed using VILBER LOURMAT (Torcy, Paris, France).

### 2.5. Immunofluorescence Assay

HUVECs were seeded onto flame-sterilized coverslips that were placed in a six-well tissue culture plate. The cells were incubated with or without ginkgolide B for 1 h, and then 0.1 mg/mL ox-LDL was added to the wells for 6 h. The cells were fixed for 15 min in 4% (w/v) paraformaldehyde/phosphate-buffered saline (PBS) and made permeable by the addition of 0.2% Triton X-100/PBS for 15 min. Blocking solution was added at 4°C for 2 h. After washing three times, anti-JAM-A, anti-Cx43, and anti-VE-cadherin antibodies were added to each well overnight. After washing three times, secondary antibodies conjugated with fluorescein isothiocyanate were added and incubated for 1.5 h. The cells were then incubated with 4′,6-diamidino-2-phenylindole (DAPI) for 5 min to stain the nucleus. The cells were imaged using a fluorescence microscope (BX60, Olympus Japan) and charge-coupled device system.

### 2.6. Monocyte Transendothelial Cell Migration Assay

Transmigration assays were performed using Transwell filters (6.5 mm diameter, 8 *μ*m pore size). Briefly, endothelial cells were grown in a collagen-coated Transwell chamber and treated with ox-LDL for 6 h, and then 2 × 10^5^ U937 monocytes were added to the Transwell chamber for 24 h. The cells were fixed with 4% paraformaldehyde for 10 min, and 0.5% cresyl violet was added to the Transwell chamber to stain monocytes for 30 min. Transmigrated monocytes were counted in five arbitrary images under a microscope.

### 2.7. JAM-A, Cx43, and VE-Cadherin Small-Interfering RNA

HUVECs were cotransfected with effectene transfection reagent (QIAGEN) that carried JAM-A, Cx43, and VE-cadherin selective small-interfering RNA (siRNA) for 72 h. The JAM-A siRNA sequence was 5′-GAAGUGAAGGAGAAUUCAA(dTdT)-3′. The Cx43 siRNA sequence was 5′-AAAGGAAGAGAAACTGAACAA(dTdT)-3′. The VE-cadherin siRNA sequence was 5′-ACGTATTATCACAATAACGAA(dTdT)-3′. HUVECs were treated with si JAM-A, si Cx43, and si VE-cadherin for 48 h, respectively. Immunoblotting was performed to examine the efficiency of protein knockdown. Transfected cells were treated with ox-LDL for 6 h, and monocytes were added to the Transwell chamber for 24 h. The monocyte transmigration assay was the same as above.

### 2.8. Statistical Analysis

All of the data represent the mean of at least three independent experiments and are expressed as mean ± SE. Statistical significance was determined by analysis of variance (ANOVA) followed by the Tukey's* post hoc* test. Differences were considered significant at values of *p* < 0.05.

## 3. Results

### 3.1. Effects of Ginkgolide B on JAM-A Expression in ox-LDL-Treated HUVECs

**Figure 1:**
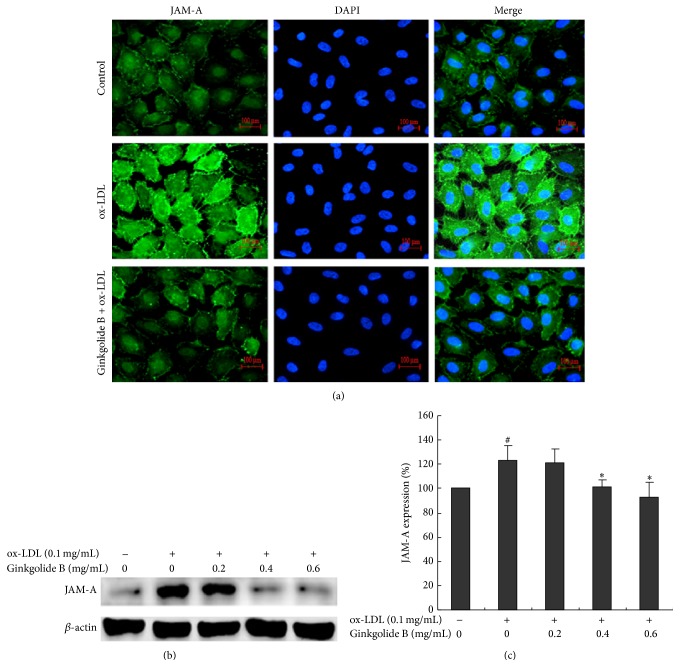
Ginkgolide B inhibits JAM-A expression in ox-LDL-treated HUVECs. (a) Immunofluorescent detection of JAM-A expression. (b) Western blot analysis of JAM-1 expression. (c) Density analysis of JAM-A expression. The data were obtained from four independent experiments. ^#^ *p* < 0.05, ox-LDL-treated cells* versus* non-ox-LDL-treated cells; ^*∗*^ *p* < 0.05, ox-LDL-treated cells* versus* ginkgolide B-treated cells.

JAM-A is a unique tight junction transmembrane protein that maintains endothelial cell-cell interactions. Under pathological conditions, JAM-A is a leukocyte adhesion molecule [26]. We performed immunofluorescence to investigate whether ginkgolide B exerts an effect on ox-LDL-induced JAM-A expression. The immunofluorescence results showed that ox-LDL stimulation (0.1 mg/mL for 6 h) enhanced JAM-A expression, and ginkgolide B (0.6 mg/mL) attenuated ox-LDL-induced JAM-A expression and returned the fluorescence intensity of JAM-A to baseline levels (Figure 1(a)). The Western blot results showed that ox-LDL (0.1 mg/mL) stimulation increased JAM-A protein expression by 35%. Pretreatment with ginkgolide B (0.2, 0.4, and 0.6 mg/mL) dose-dependently reduced ox-LDL-induced JAM-A expression. At a concentration of 0.6 mg/mL, ginkgolide B nearly completely abolished the enhancement of JAM-A expression (Figure 1(b)).

### 3.2. Effects of Ginkgolide B on Cx43 Expression in ox-LDL-Treated HUVECs

**Figure 2:**
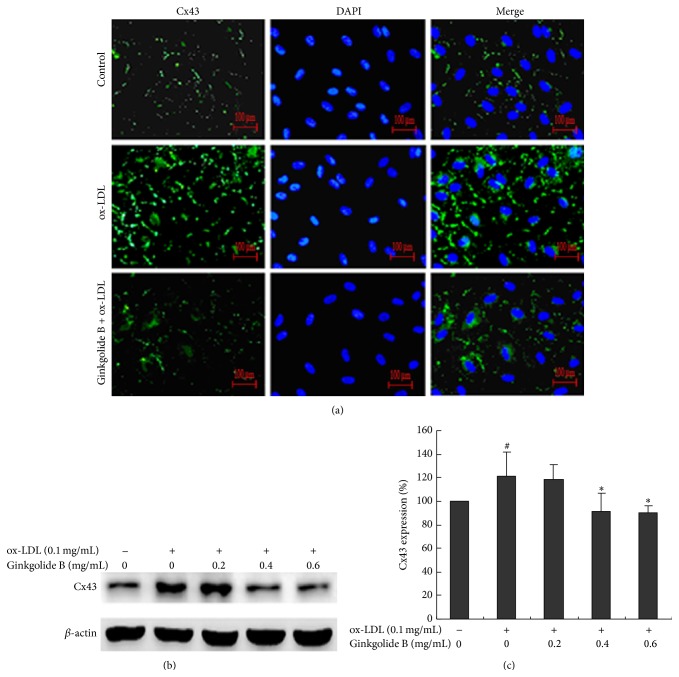
Ginkgolide B reduces Cx43 expression in ox-LDL-treated HUVECs. (a) Immunofluorescent detection of Cx43 expression. (b) Western blot analysis of Cx43 expression. (c) Density analysis of Cx43 expression. The data were obtained from four independent experiments. ^#^ *p* < 0.05, ox-LDL-treated cells* versus* non-ox-LDL-treated cells; ^*∗*^ *p* < 0.05, ox-LDL-treated cells* versus* ginkgolide B-treated cells.

We then evaluated the effect of ginkgolide B on Cx43 expression in ox-LDL-treated endothelial cells. Immunofluorescence showed that Cx43 expression was markedly enhanced in ox-LDL-treated cells. Ginkgolide B (0.6 mg/mL) almost fully abolished Cx43 expression (Figure 2(a)). The western blot analysis showed that Cx43 expression increased by 24% in ox-LDL-treated cells, and ginkgolide B (0.4 and 0.6 mg/mL) dose-dependently reduced Cx43 expression (Figures 2(b) and 2(c)).

### 3.3. Effects of Ginkgolide B on VE-Cadherin Phosphorylation and Expression in ox-LDL-Treated HUVECs

**Figure 3:**
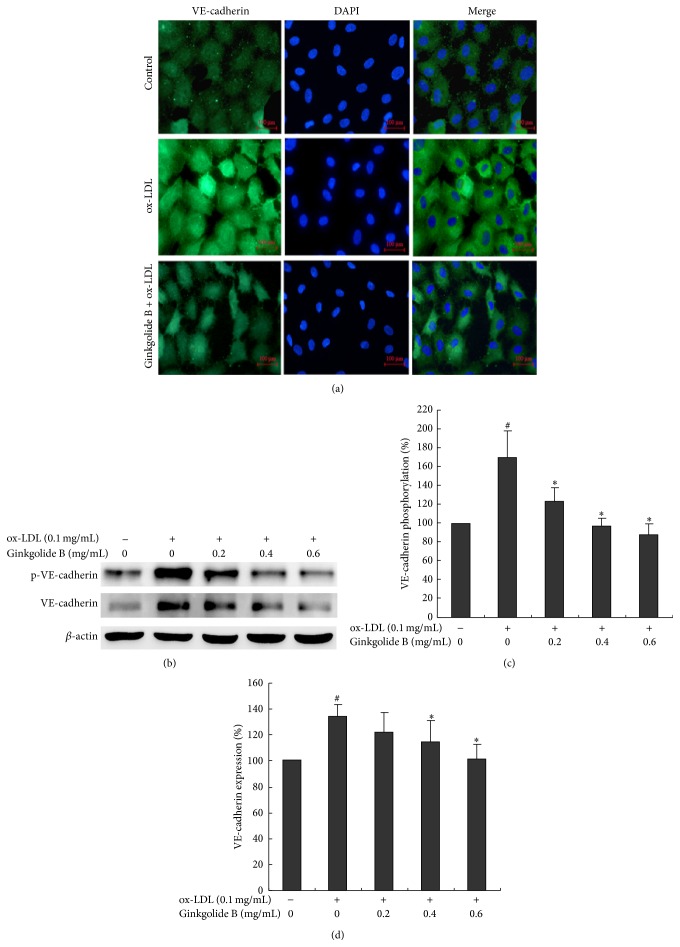
Ginkgolide B suppresses VE-cadherin expression and phosphorylation in ox-LDL-treated HUVECs. (a) Immunofluorescent detection of VE-cadherin expression. (b) Western blot analysis of VE-cadherin expression and phosphorylation. (c) Density analysis of VE-cadherin phosphorylation. (d) Density analysis of VE-cadherin expression. The data were obtained from four independent experiments. ^#^ *p* < 0.05, ox-LDL-treated cells* versus* non-ox-LDL-treated cells; ^*∗*^ *p* < 0.05, ox-LDL-treated cells* versus* ginkgolide B-treated cells.

VE-cadherin is enriched in endothelial cells and essential for controlling endothelial monolayer permeability and angiogenesis [27]. Immunofluorescence showed that VE-cadherin expression was higher in ox-LDL-stimulated cells than in control cells. Ginkgolide B treatment resulted in VE-cadherin expression that remained similar to control levels (Figure 3(a)). VE-cadherin protein expression and phosphorylation were then analyzed by Western blot. The results showed that VE-cadherin phosphorylation and expression increased by 68% and 37%, respectively, in ox-LDL-stimulated cells. In contrast, ginkgolide B (0.6 mg/mL) completely attenuated the enhanced expression and phosphorylation of VE-cadherin in ox-LDL-stimulated cells (Figures 3(b)–3(d)).

### 3.4. Effects of Ginkgolide B on Monocyte Transmigration in ox-LDL-Treated HUVECs

**Figure 4:**
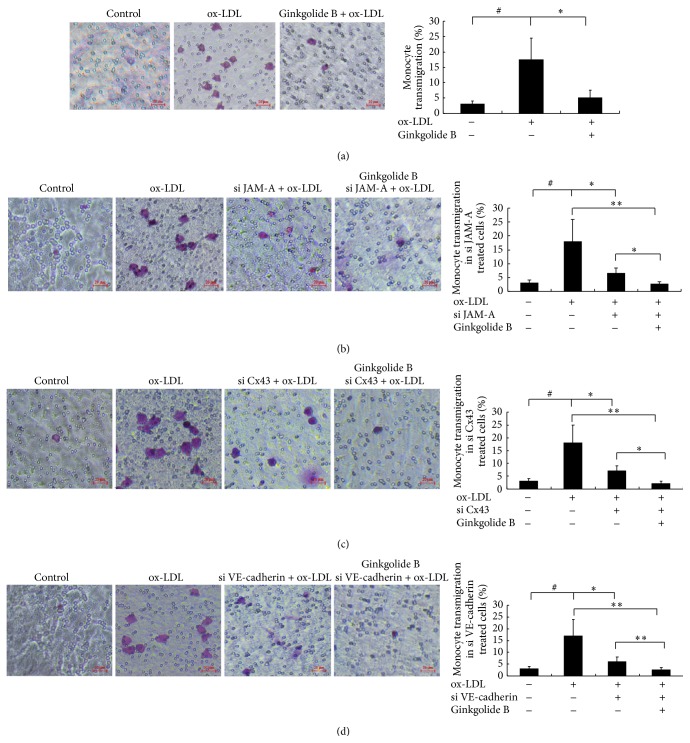
Ginkgolide B reduces monocyte transmigration in ox-LDL-treated HUVECs. (a) Ginkgolide B reduced monocyte transmigration in ox-LDL-treated endothelial cells. (b) HUVECs were treated with si JAM-A for 48 h. Monocyte transmigration was detected in ox-LDL-treated and si JAM-A-transfected cells. (c) Monocyte transmigration was detected in ox-LDL-treated and si Cx43-transfected cells. (d) Monocyte transmigration was detected in ox-LDL-treated and si VE-cadherin-transfected cells. The data were obtained from three independent experiments. ^#^ *p* < 0.05, ox-LDL-treated cells* versus* non-ox-LDL-treated cells; ^*∗*^ *p* < 0.05 and ^*∗∗*^ *p* < 0.001, ox-LDL-treated cells* versus* ginkgolide B-treated cells.

Growing evidence indicates that ox-LDL is an inducer of vascular inflammation, which leads to TJ dysfunction in atherosclerosis. However, unknown is whether ginkgolide B reduces leukocyte transmigration. In the present study, monocyte transmigration was evaluated using the Transwell assay. As shown in Figure 4(a), ox-LDL increased monocyte transmigration. The level of transmigrated monocytes was about sixfold higher in the ox-LDL-treated group than in the control group. The number of monocytes that transmigrated in ginkgolide B-treated cells was similar to controls. To characterize the role of JAM-A, Cx43, and VE-cadherin in monocyte transmigration in ox-LDL-treated cells, we used siRNA of JAM-A, CX43, and VE-cadherin to knock down the expression of these proteins. As shown in Figures 4(b)–4(d), ox-LDL-induced monocyte transmigration was markedly decreased in JAM-A siRNA-, Cx43 siRNA-, and VE-cadherin siRNA-transfected cells. Ginkgolide B synergistically inhibited monocyte transmigration with JAM-A siRNA, Cx43 siRNA, and VE-cadherin siRNA in ox-LDL-stimulated cells.

### 3.5. Effects of LY294002 on JAM-A, Cx43, and VE-Cadherin Expression in ox-LDL-Treated HUVECs

**Figure 5:**
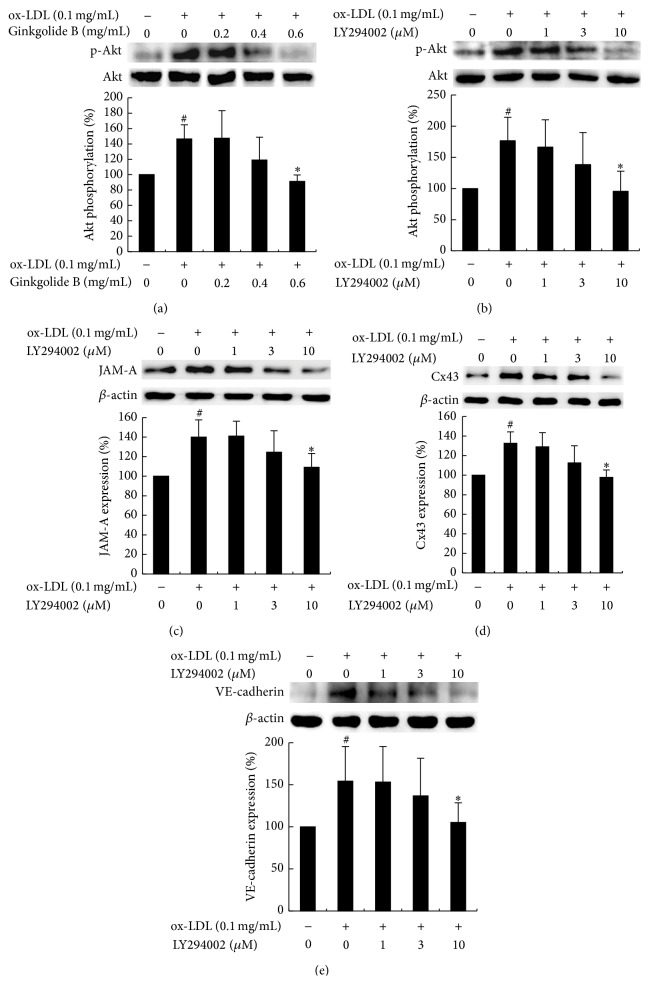
Ginkgolide B and LY294002 inhibit Akt phosphorylation and LY294002 suppresses JAM-A, Cx43, and VE-cadherin expression in ox-LDL-treated HUVECs. (a) The upper panel showed that ginkgolide B inhibits Akt phosphorylation. The lower panel is density analysis of Akt phosphorylation. (b) The upper panel showed that LY294002 inhibits Akt phosphorylation. The lower panel is density analysis of Akt phosphorylation. (c) The upper panel showed LY294002 diminishes JAM-A expression. The lower panel is density analysis of JAM-A expression. (d) The upper panel showed that LY294002 diminishes Cx43 expression. The lower panel is density analysis of Cx43 expression. (e) The upper panel showed LY294002 decreases VE-cadherin expression. The lower panel is density analysis of VE-cadherin expression. The data were obtained from three independent experiments. ^#^ *p* < 0.05, ox-LDL-treated cells* versus* non-ox-LDL-treated cells; ^*∗*^ *p* < 0.05, ox-LDL-treated cells* versus* ginkgolide B-treated cells.

We recently reported that ginkgolide B decreased inflammatory protein expression by inhibiting Akt phosphorylation in ox-LDL-treated endothelial cells [28]. In the present study, we further investigated whether ginkgolide B decreases the expression of JAM-A, Cx43, and VE-cadherin in ox-LDL-stimulated endothelial cells through the PI3K/Akt pathway. LY294002 is a specific inhibitor of PI3K and can potently suppress Akt phosphorylation. As shown in Figures 5(a) and 5(b), both ginkgolide B and LY294002 dose-dependently attenuated Akt phosphorylation that was induced by ox-LDL. The effect of LY294002 on junction protein expression was then determined. As shown in Figures 5(c)–5(e), LY294002 (1, 3, and 10 *μ*M) dose-dependently deceased JAM-A, Cx43, and VE-cadherin expression in ox-LDL-treated HUVECs.

## 4. Discussion

Increasing evidence indicates that endothelial barrier dysfunction increases vascular permeability in atherosclerosis. However, the mechanism of action of junction proteins in the increase in vascular permeability remains to be fully understood. In the present study, we provided evidence that ox-LDL stimulation increased the expression of the junction proteins JAM-A, Cx43, and VE-cadherin and promoted monocyte transmigration. We also found that ginkgolide B reduced the expression of these junction proteins and monocyte transmigration in ox-LDL-treated HUVECs.

JAM-A mediates leukocyte adhesion and transmigration via heterophilic interactions with the *α*L*β*2 integrin LFA-1 [29]. Woodfin et al. showed that endothelial cell activation leads to neutrophil transmigration that is supported by ICAM-2, JAM-A, and PECAM-1 [30]. Moreover, increases in JAM-A expression were found in human plaque biopsies and plaque in aortas in mice [29]. Schmitt et al. recently reported that impairments in JAM-A expression in endothelial cells reduced mononuclear cell recruitment into the arterial wall and limited atherosclerotic lesion formation in hyperlipidemic mice [31]. These studies revealed that JAM-A plays an important role in the modulation of vascular permeability in atherosclerosis.

Cx43 belongs to the gap junction family. Gap junction proteins have recently been implicated as important for the regulation of cell adhesion and migration in a variety of cell types. Once cells are activated, they may open and allow the exchange of ions, small metabolites, second messengers, linear peptides, or small silencing RNA while they dock with another connexin from neighboring cells [32]. This association between two connexins occurs via noncovalent interactions between extracellular loops and permits the formation of an intercellular gap junction channel. Connexins are classically considered to act as a gap junction channel-forming protein only. However, there is growing evidence that connexins exert functions in the cell that are independent of their channel-forming capacity [33]. Cx43 expression has been shown to correlate with an increase in the migration of several cell types, such as glioma cells, neural crest cells, endothelial cells, and endothelial progenitor cells [34]. Our results showed that ox-LDL stimulation increased Cx43 expression, suggesting that Cx43 might be associated with monocyte transmigration under pathological conditions.

VE-cadherin is an endothelium-specific adhesion molecule that controls the permeability of the blood vessel wall. Although VE-cadherin plays a key role in maintaining endothelial cell integrity, it also responds to inflammation, reflected by both its phosphorylation and expression. We found that the phosphorylation and expression of VE-cadherin were enhanced in ox-LDL-treated endothelial cells. In turn, VE-cadherin phosphorylation and expression were attenuated by ginkgolide B treatment.

Our results showed that JAM-A, Cx43, and VE-cadherin exhibited similar patterns in ox-LDL-stimulated endothelial cells. Moreover, the monocyte transmigration assay suggested that ox-LDL increased monocyte transmigration, and ginkgolide B treatment suppressed this process. Increases in JAM-A, Cx43, and VE-cadherin expression might mediate monocyte transmigration in ox-LDL-stimulated endothelial cells, which occurs independently of their intracellular and between-cell junction functions. To further investigate the role of JAM-1, Cx43, and VE-cadherin in monocyte transmigration, we used siRNA to knock down JAM-A, Cx43, and VE-cadherin protein expression. The results indicated that the downregulation of JAM-A, Cx43, and VE-cadherin expression led to a decrease in ox-LDL-induced monocyte transmigration. These results also imply that JAM-A, Cx43, and VE-cadherin mediate monocyte transmigration. Moreover, we found that the ginkgolide B-induced inhibition of JAM-A, Cx43, and VE-cadherin expression is linked to the suppression of Akt phosphorylation. Akt might be an important pharmacological target of ginkgolide B in the regulation of junction protein expression and amelioration of vascular permeability.

In conclusion, ginkgolide B decreased the expression of JAM-A, Cx43, and VE-cadherin and reduced monocyte transmigration in ox-LDL-treated HUVECs. These effects might be associated with improvements in vascular permeability. The mechanism of action appears to be linked to the attenuation of Akt phosphorylation in ox-LDL-stimulated HUVECs.
